# ARE CAREGIVERS HEALTHIER?: ASSESSING CAREGIVERS' EPISODIC MEMORY IN A MATCHED AND UNMATCHED SAMPLE

**DOI:** 10.1093/geroni/igac059.2077

**Published:** 2022-12-20

**Authors:** Britney Veal, Jessica Yauk, Hongdao Meng

**Affiliations:** University of South Florida, Tampa, Florida, United States; University of South Florida, Tampa, Florida, United States; University of South Florida, Tampa, Florida, United States

## Abstract

Recent findings using an advanced methodological technique of propensity matching have found that caregivers may have better cognitive health compared to non-caregivers. However, there are limited studies assessing how personality and other psychosocial variables may affect the relationship between caregiver status and cognition. Utilizing the healthy caregiver hypothesis (HCH), the current study examined the association between caregiving and episodic memory in a matched (N= 1,246) and unmatched (N=3,112) sample of caregivers from the 2016 wave of the Health and Retirement Study. The interaction between caregiving status and personality was also examined. Unadjusted models showed no difference between caregiver status and episodic memory in the samples; however, depression was significantly (p=<.0001) related to cognition in the unmatched sample. In adjusted models for the unmatched sample, conscientiousness (p=0.043), pessimism (p=0.006), and feeling constrained (p=0.028) were found to be significantly associated with episodic memory. In the matched adjusted models, conscientiousness was no longer a significant predictor, but number of chronic conditions was significantly related to episodic memory (p=0.001). The interaction between caregiving and extraversion also approached significance (p=0.076). Findings suggest extraverted caregivers may have better episodic memory performance. These findings highlight the importance of implementing propensity matching in caregiving research. Future research is needed to examine the relationship between coping style and personality specific domains in relation to the HCH.

